# Metabolic syndrome and insulin resistance in relation to biliary tract cancer and stone risks: a population-based study in Shanghai, China

**DOI:** 10.1038/bjc.2011.363

**Published:** 2011-09-13

**Authors:** F M Shebl, G Andreotti, T E Meyer, Y-T Gao, A Rashid, K Yu, M-C Shen, B-S Wang, T-Q Han, B-H Zhang, F Z Stanczyk, A W Hsing

**Affiliations:** 1 Division of Cancer Epidemiology and Genetics, National Cancer Institute, National Institutes of Health, Rockville, MD, USA; 2 Department of Epidemiology, Shanghai Cancer Institute, Shanghai, China; 3 Department of Pathology, MD Anderson Cancer Center, Houston, TX, USA; 4 Shanghai Tumor Hospital, Fudan University, Shanghai, China; 5 Zhongshan Hospital, Fudan University, Shanghai, China; 6 Ruijn Hospital, Shanghai Jiaotong University School of Medicine, Shanghai, China; 7 Institute of Oriental Hepatobiliary Surgery, Second Military Medical University, Shanghai, China; 8 Departments of Obstetrics and Gynecology and Preventive Medicine, Keck School of Medicine, University of Southern California, Los Angeles, CA, USA

**Keywords:** metabolic syndrome, insulin resistance, biliary tract cancers, biliary stones

## Abstract

**Background:**

Serum lipids, diabetes, and obesity, individual components of metabolic syndrome, are associated with biliary tract cancer and stone risk, but the associations of metabolic syndrome or insulin resistance with biliary tract cancers and stones are not well studied.

**Methods:**

In this population-based case-control study in Shanghai, China (627 biliary tract cancers, 1037 biliary stones, and 959 controls), metabolic syndrome was defined as the presence of any three of the five components, including high waist circumference, high triglycerides, low high-density lipoprotein cholesterol (HDL), high blood pressure, and diabetes. Insulin resistance and *β*-cell function were assessed, using homeostasis assessment models.

**Results:**

Metabolic syndrome was significantly associated with gallbladder cancer (odds ratio (OR)=2.75, 95% confidence interval (95% CI)=1.82–4.15) and biliary stones (OR=1.64, 95% CI=1.24–2.16), with a significant dose effect with increasing number of metabolic syndrome components (*P* trend <0.0001). The observed association persisted among subjects without a history of diabetes. The association between insulin resistance and gallbladder cancer was borderline (*P* trend=0.06). There was a significant inverse association between *β*-cell function and gallbladder cancer risk (*P* trend <0.001).

**Conclusion:**

Our findings suggest that metabolic syndrome and insulin resistance have a role in the aetiology of biliary tract cancers and biliary stones, and if confirmed, they imply that lifestyle control of these factors may lower the risk of biliary stones and biliary tract cancer.

Biliary tract cancers, including cancers of the gallbladder, extrahepatic bile duct, and ampulla of Vater, are rare but fatal malignancies (Hsing *et al*, 2006). Gallstones are undoubtedly the most important risk factor for biliary tract cancer (Hsing *et al*, 2006). Gallstones and biliary tract cancers have several risk factors in common, including obesity, diabetes, and hyperlipidaemia (Hsing *et al*, 2007, 2008; Ishiguro *et al*, 2008; Shebl *et al*, 2010), but the biologic mechanisms underlying the associations of obesity, diabetes, and hyperlipidaemia with gallstones and biliary tract cancers are not clear. These factors are components of metabolic syndrome, which is a cluster of related conditions (i.e., abdominal obesity, high blood pressure, high plasma glucose, high triglycerides, and decreased high-density lipoprotein cholesterol (HDL); (Cowey and Hardy, 2006)). While individual components of metabolic syndrome have been linked to biliary tract cancers and biliary stones (Maclure *et al*, 1989; Kodama *et al*, 1999; Tsai *et al*, 2004; Andreotti *et al*, 2008; Seidell, 2010), the total effect of metabolic syndrome on these two conditions has not been investigated fully.

To clarify further the role of metabolic syndrome in biliary tract cancer and biliary stones, we adapted a risk prediction approach to examine the associations of individual components and the collection of several components of metabolic syndrome with the risk of biliary tract cancers and biliary stones in a population-based case-control study conducted in Shanghai, China. Further, since insulin resistance is an early process of metabolic syndrome, we also examined markers of insulin resistance (fasting serum levels of glucose and insulin, derived *β*-cell function (HOMA2-%*β*), and homeostasis insulin resistance (HOMA2-%R)) with biliary tract cancer and stone risk in the same population.

## Materials and methods

### Study population

Details of the study design and population have been reported elsewhere (Hsing *et al*, 2007, 2008). Briefly, between 1997 and 2001, patients newly diagnosed with primary biliary tract cancers (ICD9 code 156) were recruited using a rapid-reporting system in 42 hospitals in Shanghai. More than 95% of incident biliary tract cancer patients in Shanghai were enrolled into the study and ∼70% of cancer cases were confirmed by histopathologic assessment or medical records, and 30% were confirmed by clinical and imaging data. Biliary stone status among cancer cases was determined based on medical record review, self-report, or clinical evaluation (ultrasound and endoscopic retrograde cholangiopancreatography). Biliary stone patients without a history of cancer were identified from the same hospitals as cancer cases, confirmed using abdominal ultrasound, medical or surgical records, or pathologic specimens, and frequency-matched to the index cancer case on diagnosing hospital, age (5-year groups), and gender. Healthy subjects without biliary tract cancer were randomly selected from the Shanghai Resident Registry, and were frequency-matched to the index cancer case on gender and age (5-year groups). Biliary stone status among population controls was based on abdominal ultrasound (85% gave consent) or self-report. Response rates for interviews were 95% among cases and 82% among eligible controls.

### Interview

In-person interviews were conducted by trained interviewers using a structured questionnaire to obtain information on demographic characteristics, lifestyle factors, and medical conditions. Cancer and stone cases were interviewed within 3 weeks of diagnosis. Five percent of the subjects were re-interviewed 3 months after the initial interview to assess the reliability of the self-reported data. Concordance between the two interviews was >90%.

At interview, weight, height, and waist and hip circumference were measured. Physical measurements were taken twice. When the difference between the two measurements exceeded *a priori* tolerance limits (height, 2 cm; weight, 1 kg; waist and hip circumference, 2 cm), an additional measurement was taken and the average of the closest two values was used. We also used self-reported height and weight to calculate usual adult body mass index (BMI; kg m^−2^) 5 years before interview. Diabetes and hypertension were based on self-reported information.

### Biologic sample collection and assessment

Overnight fasting blood samples were collected from over 80% of the participants who gave consent. Within 4 h of collection, study personnel transported the samples to the central processing laboratory at the Shanghai Cancer Institute for processing, and samples were later shipped to the NCI repository on dry ice via Federal Express. Serum lipids were measured at the Laboratory of Biochemistry, Institute of Cardiovascular Diseases, Zhongshan Hospital, Shanghai Medical University (Fudan University). Assay kits for total cholesterol (oxidation enzymatic assay) (Smith *et al*, 1979), triglycerides (glycerol phosphoric acid oxidase assay) (Smith *et al*, 1979), and HDL (phosphotungstate-magnesium assay) (National Institutes of Health, 1995) were prepared by Shanghai No. 18 Pharmaceutical Company and used the spectrophotometer type 722 (Shanghai Third Analysis Instrument Company, Shanghai, China). LDL was calculated using the Friedewald formula (Rifai *et al*, 1992). Fasting blood glucose and human specific insulin were measured using commercially available radioimmunoassay kits (Linco Research, St Charles, MO, USA) at the University of Southern California (FZS).

### Metabolic syndrome and insulin resistance components

Metabolic syndrome was defined based on the criteria established by the Chinese Joint Committee for Developing Chinese Guidelines on Prevention and Treatment of Dyslipidemia in Adults (2007), which is similar to the criteria used by the American Heart Association/National Heart, Lung, and Blood Institute (Grundy *et al*, 2005), although the cutoffs are based on the distribution in the Chinese population and are lower than those used for Americans. An individual is classified as having metabolic syndrome if he/she has any three of these five conditions: high triglycerides (⩾1.7 mmol l^−1^), low HDL (<1.04 mmol l^−1^ for both men and women), high blood pressure (self-reported), diabetes (self-reported), and high waist circumference (⩾90 cm in men and ⩾80 cm in women).

For analyses of insulin resistance, we evaluated fasting serum levels of glucose and insulin, and using these measurements we derived a homeostasis assessment model 2 (HOMA2) for *β*-cell function (HOMA2-%*β*) and insulin resistance (HOMA2-%R) (models were estimated using software that is available at http://www.dtu.ox.ac.uk/index. php?maindoc=/homa/; Levy *et al*, 1998). The HOMA2 is a method used to quantify insulin resistance and *β*-cell function. The higher the levels of HOMA2-%R the higher the insulin resistance. The higher the levels of HOMA2-%*β* the higher the insulin sensitivity.

### Statistical analyses

We examined each of the five components of metabolic syndrome individually, the total effect of metabolic syndrome (any three of the five components), and the degree of metabolic syndrome by calculating a *P* trend for the number of components ranging from 0 to 5 factors.

Measurements of the insulin sensitivity markers were categorised into tertiles based on the distribution in the non-diabetic control population. Correlations between log-transformed insulin sensitivity markers were calculated using Spearman correlation coefficients. *T*-tests and Kruskal–Wallis statistics (when the assumption of normality was violated) were used for the bivariate comparison of cases and controls.

For all analyses, bile duct and ampulla of Vater cancers were compared with all population controls, gallbladder cancers were compared with population controls who did not have a cholecystectomy, and biliary stones cases were compared with population controls who did not have stones. All analyses were conducted using SAS 9.1 (SAS Institute, Cary, NC, USA). A total of 368 gallbladder, 191 extrahepatic bile duct, and 68 ampulla of Vater cancer cases, as well as 1037 patients with biliary stones, and 959 population controls were included in the metabolic syndrome analysis. The analysis of insulin resistance was restricted to subjects who donated blood samples and did not have a history of diabetes (self-reported) since diabetes and anti-diabetic medications can influence insulin sensitivity indices; this resulted in 219 cases with gallbladder cancer, 116 with extrahepatic bile duct cancer, with 926 biliary stones, and 553 population controls. Cases were frequency-matched; therefore, we used unconditional multivariable logistic regression to calculate odds ratios (ORs) and 95% confidence intervals (95% CIs) adjusted for age, gender, and BMI. Body mass index was grouped according to the WHO classification for Asian populations where 18.5 to <23 kg m^−2^ is considered normal, 23 to <25 kg m^−2^ overweight, and ⩾25 kg m^−2^ obese (WHO/IASO/IOTF, 2000; WHO Expert Consultation, 2004). To reduce the impact of cancer-related wasting, we excluded individuals who reported >10% weight loss during the 5 years before cancer diagnosis.

## Results

Associations of individual metabolic syndrome components with biliary tract cancer and biliary stone risk are shown in Table 1. Results for the ampulla of Vater cancers are not included due to small numbers. After adjustment for age, gender, and BMI, high serum triglycerides (⩾1.7 mmol l^−1^) were associated with excess risks of gallbladder cancer (OR=2.0, 95% CI=1.48–2.70), extrahepatic bile duct cancer (OR=5.28, 95% CI=3.56–7.82), and biliary stones (OR=1.56, 95% CI=1.24–1.98), while low serum HDL was associated with excess risks of gallbladder cancer (OR=7.53, 95% CI=5.36–15.59), extrahepatic bile duct cancer (OR=8.17, 95% CI=5.17–12.92), and biliary stones (OR=3.12, 95% CI=2.50–3.89). Diabetes (self-reported) was also associated with excess risks of gallbladder cancer and biliary stones (OR=1.95, 95% CI=1.18–2.60; OR=1.69, 95% CI=1.17–2.44, respectively). Hypertension had a significant inverse association with all three biliary diseases, while the effect of waist circumference was null.

Metabolic syndrome (any three of the five components) was significantly associated with increased risks of all three cancers: gallbladder cancer (OR=2.75, 95% CI=1.82–4.15), bile duct cancer (OR=1.92, 95% CI=1.07–3.42), and biliary stones (OR=1.64, 95% CI=1.24–2.16), after adjustment for age, gender, and BMI (Table 1). Similar risk estimates persisted when the analysis was restricted to individuals with BMI<25 or non-diabetics (metabolic syndrome defined as any three of the remaining four components). The risks of gallbladder cancer, bile duct cancer, and biliary stones increased with increasing number of metabolic syndrome components (*P* for trend <0.0001), with those having 4–5 components having risks as high as 4.5-fold when compared with those having 0–1 components (Table 1). Similar risk estimates were seen, regardless of BMI status, or gallstone status. We observed more pronounced associations in women than in men.

Associations for markers of insulin resistance among individuals without a history of diabetes are shown in Table 2. After adjustment for age, gender, and BMI, high fasting glucose was significantly associated with significant excess risk of gallbladder and bile duct cancer and biliary stones. Participants in the highest tertile of HOMA2-%*β* (least insulin resistant) had a reduced risk of gallbladder cancer (OR=0.38, 95% CI=0.24–0.58), bile duct cancer (OR=0.38, 95% CI=0.21–0.67), and biliary stones (OR=0.70, 95% CI=0.51–0.97) compared with individuals in the lowest tertile of HOMA2-%*β* (most insulin resistant). Fasting insulin and HOMA2-%R were not significantly associated with biliary tract cancers or stones.

Correlations among the metabolic and insulin sensitivity biomarkers were high among non-diabetic controls (Supplementary Table 1). HOMA2-%*β* was positively correlated with BMI and stones. HOMA2-%R was positively correlated with BMI, waist circumference, and stones. Serum levels of insulin and glucose were positively correlated with BMI, waist circumference, and stones.

## Discussion

In this population-based study, metabolic syndrome and insulin resistance (among those without a history of diabetes) were associated with excess risks of biliary tract cancer and stones. There was a dose-response relationship between increasing number of metabolic syndrome components and risk of biliary tract cancer and stones. The observed association between metabolic syndrome and biliary tract cancer and stones was more pronounced in women than in men, and it persisted among non-obese (BMI<25) and non-diabetic individuals.

Interestingly, the association between some components of metabolic syndrome, such as HDL, and cancer was stronger than that between metabolic syndrome (defined as having three or more of the five components) and cancer. This results because the definition of metabolic syndrome is stricter than the definition of the individual components, so that individuals have to have three or more components to be classified as having metabolic syndrome. Cancer patients who have only one or two of the components would not be classified as having metabolic syndrome. Therefore, the prevalence of metabolic syndrome is lower than that of each individual component in cancer patients, resulting in smaller ORs for metabolic syndrome than for the individual components.

The observed association with metabolic syndrome does not appear to be explained entirely by a single component of the syndrome, since there was a significant dose-response relationship between the number of metabolic syndrome components and the risk of biliary tract cancer and stones. This dose-response relationship suggests that the presence of multiple components may have an additive effect on the risk of biliary tract cancers and stones. It is noteworthy that, although only three of the five individual components were positively associated with stone and cancer risks in the study, individuals having four or five components had the highest risk (4.5-fold). This observation suggests that the severity of metabolic syndrome exacerbates the development of stones and cancer.

The association of metabolic syndrome with biliary tract cancer and stones is expected and biologically plausible, as several of the components have been individually linked to stone and cancer risk in the study. For example, earlier we reported that, independent of BMI, diabetes was associated with a 2-fold risk of biliary stone and cancer, with biliary stones and low HDL accounting for 60% and 17% of the diabetes effect on gallbladder cancer risk (Shebl *et al*, 2010). It has also been shown that low serum HDL and high triglycerides result in oversaturation of cholesterol in bile and impaired gallbladder emptying, which leads to the formation of gallstones (Janowitz *et al*, 1992; Jonkers *et al*, 2003; Shor *et al*, 2008; Maurer *et al*, 2009; Tabet and Rye, 2009), and thereby to a higher risk of biliary tract cancer. Independent of stone formation, low serum HDL and high triglycerides are associated with increased LDL oxidation and formation of reactive oxygen species (ROS; Shor *et al*, 2008; Tabet and Rye, 2009), which in turn can increase oxidative DNA damage and promote cancer development (Jaiswal *et al*, 2001). Although hypertension was not individually positively associated with gallbladder cancer risk, the clustering of hypertension with other four components of metabolic syndrome was associated with a 4-fold risk. The inverse association with hypertension needs to be replicated.

The lack of an association with waist circumference was somewhat surprising, but could be explained in part by the inherent limitation of the cross-sectional design of the study. Waist circumference in our study was measured at interview, which may have been affected by the presence of cancer (and weight loss), and may not represent usual adult waist size. Similarly, in our earlier analysis, BMI at interview was not associated with biliary tract cancer or stones, while usual adult BMI (5 years before interview) was associated with an excess risk (Hsing *et al*, 2008), suggesting that body size at interview (closer to the time of cancer diagnosis) may not reflect usual adult body size.

The observation that, among individuals without a history of diabetes, hyperglycaemia and impaired *β*-cell function were associated with biliary tract cancer and stone risk underscores the importance of insulin resistance, even in the pre-diabetes state. Hyperglycaemia, characterised by high fasting glucose and insulin levels, and impaired *β*-cell function are markers of insulin resistance. It has been shown that before the development of overt type 2 diabetes, insulin resistance, including conditions of hyperglycaemia, progressive deterioration of *β*-cell function, and hyperinsulinaemia, can exist for many years in a pre-diabetic stage (Lyssenko *et al*, 2005). Hyperglycaemia, in addition to being part of insulin resistance, is associated with increased oxidative stress, accumulation of advanced glycation end products, which in turn can lead to activation of NF-*κ*B, formation of ROS, and tumourigenesis (Abe and Yamagishi, 2008).

Part of the biologic mechanism leading from insulin resistance and metabolic syndrome to the development of biliary tract cancers may be mediated by biliary stones. Adjustment for stones substantially attenuated the association between metabolic syndrome and biliary tract cancer (data not shown), suggesting that stones may mediate part of the association between metabolic syndrome and cancer. It has been shown that inflammation has a key role in stone formation as well as in the pathogenesis of metabolic syndrome and insulin resistance (de Luca and Olefsky, 2008; Maurer *et al*, 2009; Olefsky and Glass, 2010). Therefore, it is likely that chronic inflammation may act as one of the mechanisms that underlie the association of obesity, diabetes, insulin resistance, and the metabolic syndrome with stones and biliary tract cancer. It should be noted that obesity, a common risk factor for diabetes, insulin resistance, and metabolic syndrome, is considered an inflammatory disease characterised by a state of chronic subclinical inflammation, which is involved in the pathogenesis of insulin resistance in the absence of overt diabetes (Bastard *et al*, 2006).

Strengths of our study include the relatively large size and minimal selection bias due to the population-based design and high response rate. Because subject selection and response are unrelated to the exposure of interest (i.e., metabolic syndrome) selection bias in our study should be minimal. In addition, misclassification of outcome (cancer and stones) is also minimal due to the detailed evaluation of cancer status from clinical and pathology reports combined with near complete assessment of stone status through careful review of medical records and ultrasound examinations. We also minimised the impact of confounding by adjusting for known confounders, such as age, gender, and BMI, although residual confounding may still exist. An example of possible residual confounding is treatment for dyslipidaemia. However, if individuals were on treatment they would have had lower levels of triglyceride, which would bias the association towards the null. Therefore, our observed association is conservative and is an underestimate of the effect of dyslipidaemia and metabolic syndrome.

Limitations of the study should be noted. Misclassification of biomarkers among cases, such as HDL, insulin, and glucose, is possible, since blood samples were taken at enrollment; therefore, the measurements in some cases may have been affected by weight loss among cases as a result of cancer. Although we are not able to remove the effect of reverse causation entirely, we attempted to minimise its impact by limiting the analysis to those with <10% weight loss in the previous 5 years. Of note, due to the study design, we were unable to address the relationship between duration of metabolic syndrome and cancer/stone risk.

In summary, our study showed an increased risk of biliary tract cancer and biliary stones among individuals with metabolic syndrome and insulin resistance without a history of diabetes. These findings, if confirmed, suggest that lifestyle changes to control metabolic syndrome may help lower the risk of biliary stones and cancer.

## Figures and Tables

**Table 1:** Odds ratios (ORs) and 95% confidence intervals (CIs) for biliary tract cancer and biliary stones in relation to metabolic syndrome and its individual components

	**Biliary tract cancers**								
	**Gallbladder^a^**			**Extrahepatic bile duct^b^**			**Biliary stones^c^**		
	**Controls *N***	**Cases *N***	**OR (95% CI)^d^**	**Controls *N***	**Cases *N***	**OR (95% CI)^d^**	**Controls *N***	**Cases *N***	**OR (95% CI)^d^**
*Metabolic syndrome*									
*Individual components*									
Triglycerides (mmol l^−1^)									
<1.7	597	150	Ref	635	52	Ref	502	637	Ref
⩾1.7	206	114	2.00 (1.48–2.70)	223	89	5.28 (3.56–7.82)	151	344	1.56 (1.24–1.98)
HDL (mmol l^−1^)									
⩾1.04	531	58	Ref	566	26	Ref	439	378	Ref
<1.04	272	206	7.53 (5.36–15.59)	292	114	8.17 (5.17–12.92)	214	603	3.12 (2.50–3.89)
Hypertension^e^									
No	526	230	Ref	553	130	Ref	441	695	Ref
Yes	376	138	0.69 (0.53–0.90)	406	61	0.61 (0.43–0.86)	294	342	0.69 (0.56–0.86)
Diabetes^f^									
No	834	316	Ref	881	171	Ref	688	925	Ref
Yes	68	51	1.75 (1.18–2.60)	78	20	1.37 (0.81–2.33)	47	111	1.69 (1.17–2.44)
Waist circumferenceg,^h^									
Low	462	83	Ref	480	61	Ref	400	447	Ref
High	386	111	0.98 (0.65–1.47)	422	31	0.64 (0.37–1.13)	297	402	0.89 (0.69–1.16)
									
*Metabolic syndrome* h, i									
No	604	80	Ref	635	50	Ref	508	570	Ref
Yes	157	63	2.75 (1.82–4.15)	178	22	1.92 (1.07–3.42)	117	231	1.64 (1.24–2.16)
									
*Metabolic syndrome among individuals with BMI<25* h, i									
No	481	61	Ref	497	41	Ref	414	429	Ref
Yes	75	27	2.59 (1.49–4.48)	84	16	2.49 (1.28–4.85)	58	104	1.70 (1.17–2.47)
									
*Metabolic syndrome among non-diabetics* h, i									
No	585	77	Ref	614	47	Ref	493	550	Ref
Yes	124	47	2.68 (1.71–4.21)	139	22	2.56 (1.41–4.62)	94	183	1.65 (1.23–2.23)
									
*Elements of metabolic syndrome* h, j									
Total									
0–1	400	38	Ref	417	17	Ref	347	334	Ref
2	204	42	2.03 (1.24–3.34)	218	33	5.01 (2.63–9.56)	161	236	1.43 (1.09–1.88)
3	103	37	3.68 (2.13–6.36)	119	20	5.75 (2.76–12.02)	81	144	1.79 (1.27–2.51)
4–5	54	26	4.72 (2.47–9.00)	59	2	—	36	87	2.29 (1.46–3.60)
*P* trend			<0.0001			<0.0001			<0.0001
									
*Females*h,^j^									
0–1	222	26	Ref	232	6	Ref	187	206	Ref
2	132	31	2.18 (1.20–3.98)	144	10	3.78 (1.25–11.44)	99	146	1.45 (1.01–2.07)
3	65	28	4.18 (2.15–8.13)	77	10	7.74 (2.44–24.55)	47	88	2.00 (1.28–3.12)
4–5	37	21	5.49 (2.56–11.76)	40	1	—	23	66	3.20 (1.82–5.63)
*P* trend			<0.0001			<0.01			<0.0001
									
*Males*h,^j^									
0–1	178	12	Ref	185	11	Ref	160	128	Ref
2	72	11	1.98 (0.79–4.93)	74	23	5.96 (2. 76–13.28)	62	90	1.55 (1.01–2.36)
3	38	9	2.89 (1.06–7.86)	42	10	4.06 (1.55–10.64)	34	56	1.58 (0.93–2.68)
4–5	17	5	3.61 (0.97–13.42)	19	1	—	13	21	1.24 (0.56–2.75)
*P* trend			0.02			<0.01			0.11

Abbreviations: BMI=body mass index; HDL=high-density lipoprotein cholesterol.

a Gallbladder cancer cases were compared with population controls without history of prior cholecystectomy.

b Extrahepatic bile duct and ampulla of Vater cancer cases were compared with all population controls; ampulla of Vater cancer analyses are not shown due to small cell sizes.

c Individuals with gallstones were compared with controls without gallstones.

d Separate models were run for metabolic syndrome, metabolic syndrome score and different insulin resistance markers, adjusted for age, gender, and body mass index.

e Self-reported hypertension.

f Self-reported diabetes.

g Measured at interview.

h Restricted to individuals who did not lose >10% of weight within the 5 years before the interview.

i Based on Chinese Joint Committee for Developing Chinese Guidelines on Prevention and Treatment of Dyslipidemia in Adults (JCDCG) which includes the presence of any three of the five components.

j Sum of the JCDCG components (range from 0 to 5).

**Table 2:** Odds ratios (ORs) and 95% confidence intervals (CIs) for biliary tract cancer and gallstones in relation to insulin resistance/sensitivity markers among subjects without a history of diabetes

		**Biliary tract cancers**					
	**Population controls**	**Gallbladder^a^**		**Extrahepatic bile duct^b^**		**Biliary stones^c^**	
	***N*=553**	***N*=219**	**OR (95% CI)^d^**	***N*=116**	**OR (95% CI)^d^**	***N*=926**	**OR (95% CI)^d^**
*Fasting glucose (mg dl* ^−1^ *)* e							
<100	357	68	Ref	42	Ref	432	Ref
⩾100	159	125	4.49 (3.09–6.53)	66	3.92 (2.45)	302	1.84 (1.38–2.43)
*P heterogeneity*			<0.001		<0.001		<0.001
							
*Insulin (*μ*U ml*^−1^*)*^e^							
⩽5.61	170	54	Ref	38	Ref	255	Ref
>5.61 to ⩽8.40	168	57	0.99 (0.63–1.56)	32	0.80 (0.46–1.39)	220	0.80 (0.58–1.11)
>8.4	168	79	1.42 (0.91–2.23)	36	0.99 (0.56–1.74)	246	1.06 (0.76–1.49)
*P* trend			0.11		0.97		0.76
							
*HOMA2* β*-cell function (HOMA2-%*β*)*^e^							
⩽69.4	169	100	Ref	58	Ref	304	Ref
>69.4 to ⩽93.2	167	47	0.48 (0.32–0.73)	26	0.48 (0.28–0.82)	204	0.63 (0.46–0.86)
>93.2	168	43	0.38 (0.24–0.58)	22	0.38 (0.21–0.67)	213	0.70 (0.51–0.97)
*P* trend			<0.001		<0.01		0.02
							
*HOMA2 insulin resistance (HOMA2-%R)* e							
⩽0.745	167	53	Ref	35	Ref	248	Ref
>0.745 to ⩽1.112	169	55	0.97 (0.61–1.53)	30	0.86 (0.49–1.51)	231	0.87 (0.63–1.20)
>1.112	168	82	1.49 (0.95–2.34)	41	1.26 (0.72–2.22)	242	1.07 (0.76–1.50)
*P* trend			0.06		0.40		0.73

Abbreviation: HOMA2=homeostasis assessment model 2.

a Gallbladder cancer cases were compared with population controls without history of prior cholecystectomy.

b Extrahepatic bile duct cancer cases were compared with all population controls.

c Individuals with biliary stones were compared with controls without biliary stones.

d Separate models were run for metabolic syndrome, metabolic syndrome score and different insulin resistance markers, adjusted for age, gender, and body mass index.

e Analysis was restricted to non-diabetics who had enough collected blood sample.
