# Supplementary material for: Metabolic syndrome and insulin resistance in relation to biliary tract cancer and stone risks: a population-based study in Shanghai, China
Source: Br J Cancer. 2011 Sep 13;105(9):1424–9. doi: 10.1038/bjc.2011.363 (PMC3241543; doi:10.1038/bjc.2011.363)
Supplement: Supplementary Table 1 [file bjc2011363x1.doc]

| **Supplementary Table 1: Age adjusted spearman correlations coefficients between selected variables and insulin resistance among non-diabetic controls (n=502)^1,2^** | | | | |
| --- | --- | --- | --- | --- |
|  | Insulin | Glucose | HOMA2 β-cell function (HOMA2- %β)^3^ | HOMA2 insulin resistance (HOMA2-%R)^3^ |
| **Insulin** | 1.00 |  |  |  |
| **Glucose** | 0.36 <0.0001 | 1.00 |  |  |
| **HOMA2 β-cell function (HOMA2- %β)** | 0.71 <0.0001 | -0.32 <0.0001 | 1.00 |  |
| **HOMA2 insulin resistance (HOMA2-%R)** | 1.00 <0.0001 | 0.41 <0.0001 | 0.67 <0.0001 | 1.00 |
| **BMI^4^** | 0.39 <0.0001 | 0.23 <0.0001 | 0.21 <0.0001 | 0.39 <0.0001 |
| **Waist-to-hip ratio^5^** | 0.19 <0.0001 | 0.16 <0.001 | 0.06 0.20 | 0.19 <0.0001 |
| **Gallstone** | 0.19 <0.0001 | 0.07 0.10 | 0.14 <0.01 | 0.19 <0.0001 |
| **Waist circumference^6^** | 0.39 <0.0001 | 0.21 <0.0001 | 0.22 <0.0001 | 0.40 <0.0001 |
| **Triglycerides^7^** | 0.33 <0.0001 | 0.14 <0.01 | 0.22 <0.0001 | 0.33 <0.0001 |
| **HDL^8^** | 0.17 <0.001 | 0.06 0.22 | 0.13 <0.01 | 0.17 <0.001 |
| **Hypertension** | 0.12 <0.01 | 0.05 0.25 | 0.08 0.09 | 0.12 <0.01 |
| ^1^ Spearman correlations; ^2^ All insulin-related indices were included as continuous variables; ^3^ Estimated by homeostasis model assessment 2; ^4^ BMI = weight in Kg/height in meter^2^ five years before interview. Categories based on WHO classification for Asians; ^5^ Measured at interview, quartile cutoff points were based on distribution among all population controls without history of prior cholecystectomy; ^6^ Measured at interview; ^7^ Measured at interview, high triglycerides > 1.7 mmol/l; ^8^ Measured at interview, high HDL > 1.04 mmol/l | | | | |
